# Congenital *Plasmodium falciparum *infection in neonates in Muheza District, Tanzania

**DOI:** 10.1186/1475-2875-7-117

**Published:** 2008-07-03

**Authors:** Grace W Mwangoka, Sharadhuli I Kimera, Leonard EG Mboera

**Affiliations:** 1 Ifakara Health Research and Development Centre, Bagamoyo Research and Training Unit, Bagamoyo, Tanzania; 2 Sokoine University of Agriculture, Department of Veterinary Medicine and Public Health, P.O. Box 3021, Chuo Kikuu, Morogoro, Tanzania; 3 National Institute for Medical Research, Ocean Road, P.O. Box 9653, Dar es salaam, Tanzania

## Abstract

**Background:**

Although recent reports on congenital malaria suggest that the incidence is increasing, it is difficult to determine whether the clinical disease is due to parasites acquired before delivery or as a result of contamination by maternal blood at birth. Understanding of the method of parasite acquisition is important for estimating the time incidence of congenital malaria and design of preventive measures. The aim of this study was to determine whether the first *Plasmodium falciparum *malaria disease in infants is due to same parasites present on the placenta at birth.

**Methods:**

Babies born to mothers with *P. falciparum *parasites on the placenta detected by PCR were followed up to two years and observed for malaria episodes. Paired placental and infant peripheral blood samples at first malaria episode within first three months of life were genotyped (*msp2*) to determine genetic relatedness. Selected amplifications from nested PCR were sequenced and compared between pairs.

**Results:**

Eighteen (19.1%) out of 95 infants who were followed up developed clinical malaria within the first three months of age. Eight pairs (60%) out of 14 pairs of sequenced placental and cord samples were genetically related while six (40%) were genetically unrelated. One pair (14.3%) out of seven pairs of sequenced placental and infants samples were genetically related. In addition, infants born from primigravidae mothers were more likely to be infected with *P. falciparum *(*P *< 0.001) as compared to infants from secundigravidae and multigravidae mothers during the two years of follow up. Infants from multigravidae mothers got the first *P. falciparum *infection earlier than those from secundigravidae and primigravidae mothers (RR = 1.43).

**Conclusion:**

*Plasmodium falciparum *malaria parasites present on the placenta as detected by PCR are more likely to result in clinical disease (congenital malaria) in the infant during the first three months of life. However, sequencing data seem to question the validity of this likelihood. Therefore, the relationship between placental parasites and first clinical disease need to be confirmed in larger studies.

## Background

About 300,000 foetal and infants deaths and 2,500 deaths of pregnant women are attributable to malaria [1]. Poor outcome for both mother and foetus is associated with pregnancy malaria and results in premature delivery, intrauterine growth retardation, perinatal mortality, anaemia, abortion, low birth weight and death of the mother [2,3]. Moreover, perinatal malaria infection may cause cord parasitaemia, early neonatal malaria, as well as neonatal malaria. When the placenta is infected with malaria parasites, transplacental transmission of the parasites can occur, although the born infant may remain asymptomatic and healthy [4].

Congenital malaria infection refers to diagnosis of malaria parasites in the newborn within seven days of birth, or later if there is no possibility of postpartum infection by either mosquito bite or blood transfusion. On the other hand, neonatal malaria may be defined as symptoms attributed to malaria parasites in the erythrocytes of an infant within the first twenty-eight days of life [5]. The mechanism of transplacental passage of this infection is not clear. Many reports on congenital malaria have been published, showing that the incidence has recently increased. However, it is difficult from reported cases to determine whether this represents infection of the foetus before delivery or contamination by maternal blood at birth producing a replicating infection in the newborn. Infants below three months of age have been reported to have less incidence, severity, parasitaemia and death from malaria [6]. The current study was carried out to determine whether the first falciparum infection of infants within the first three months of life arose from congenitally acquired placental infection, in view of providing useful information on the understanding of the source of parasites in congenital malaria and development of immunity in infants at risk of acquiring malaria.

## Methods

### Study area

The study was conducted at Muheza District Designated Hospital, in Muheza District, north-east Tanzania. Muheza district, (5°42' S and 38°48'E) has a human population of 278,405 and the inhabitants are mainly involved in subsistence agricultural production. The district is served by one district hospital, three health centres and 32 dispensaries. The lowland areas of the districts are areas of intense transmission [7]. The entomologic inoculation rate in the area has been described as approximately 34 in the highlands and 400 infected mosquito bites each year in the lowlands [8].

### Study population

The study population comprised of mothers aged between 18 and 45 years who had placental *Plasmodium falciparum *parasites and their respective neonates. Recruitment of the study participants was done from September 2002 to September 2005 from maternity ward at Muheza DDH and 1,022 mother-neonate pairs were screened. Mothers diagnosed with placental parasitaemia identified in a blood smear by microscopy and their respective neonates were enrolled in the study. Mothers with evidence of chronic or debilitating illness, such as recent significant weight loss, chronic diarrhoea, or history of chronic illness were excluded. All infants born from mothers diagnosed with placental parasitaemia were seen at birth and at two-week intervals for two years for full clinical examination and blood sampling, either at the Muheza Designated District Hospital or at the Projects sponsored mobile clinics that rotated through study villages on a weekly basis. Written informed consent was obtained from the mother prior to recruitment into the study.

Demographic information on maternal age, village of residence, gravidity, history of intermittent presumptive therapy (IPT) against malaria during pregnancy, abortion or miscarriage;as well as baby information on birthweight and gestation age were collected by trained nurses at enrollment. Bed net usage was assessed by village health workers during clinical visits and onsite home visits. The study protocol was approved by the International Clinical Studies Review Committee of the Division of Microbiology and Infectious Diseases at the US National Institutes of Health, and ethical clearance was obtained from the Institutional Review Boards of Seattle Biomedical Research Institute and the National Institute for Medical Research in Tanzania.

### Sample collection

Blood samples of study mothers diagnosed positive for *P. falciparum *by microscopy were collected by the project staff nurses from placental tissues, umbilical cord and through either finger or heel prick of new born infants. Placental blood samples were obtained after delivery by mechanically grinding full thickness placental tissue [9]. Cord blood was collected by cannulation of the thoroughly cleaned umbilical vessels while keeping the cord clamped. Blood samples from healthy infants were collected by heel prick every two weeks and at the time of any illness through mobile clinics until when they presented with first infection. In order to determine the outcome of placental malaria, all infants from mothers diagnosed with placental parasitaemia were followed for two years. All placental samples from these mothers tested *P. falciparum *positive in their thick smears and all infants born from these mothers who presented with parasitaemia within three months from birth were selected for further analysis by PCR. In order to analyse the phenomenon of malaria infections of neonates via transmission across the placental, matched umbilical cord blood samples were examined in thick blood smear and further analysed whether negative by Giemsa stain or not. About 10 μl of blood samples were bloated on 0.5 × 2 cm strips of 3 MM Whatman filter papers and dried in a laminar flow hood. After drying, the filter papers were packed in small individual plastic bags to avoid contamination and stored at room temperature until needed for analysis.

### Extraction of parasite DNA and PCR amplification

Genomic DNA was extracted from blood collected on 3 MM Whatmann filter papers using Generation^® ^capture card kit (Gentra Systems, Minneapolis, USA). The DNA was amplified in two nested PCR using oligonucleotide primers of the 5' and 3' conserved regions as described by [10]. The details of two sets primer sequences used in the PCR for DNA amplification are shown in Table 1. PCR conditions included 94°C-1 min, 50.3°C-1 min, 72°C-45 sec., × 40, 72°C-5 min, 4°C-hold. In both reactions nest one and two PCR, reaction volumes ranged between 25 μl to 50 μl and the final concentration contained,5 mM Tris HCl (pH 8.3), 50 mM KCl, 0.5 mg of gelatin per ml, 2.5 mM MgCl_2_, 200 μM mixture of dNTPs (Pharmacia Biotech, Uppsala, Sweden), and 0.25 U/μl of *Taq *polymerase (Boehringer, Mannheim, Germany). To each PCR tubes, 5 μl of DNA was added in primary reaction and 2 μl was re- amplified in the nested PCR reaction and carried out in a PTC^®^-100 Peltier. The expected lengths of the bands were 509 bp and 750 bp and the previously established DNA positive control sample used was 3D7. In all PCR reactions a negative control sample with no template DNA was used.

**Table 1:** Sequences of the primers used for amplification MSP2 by PCR

Gene	Marker	Sequence	Location
*Msp 2*	MSP2N1 Forward 1	5'GAAGGTAATTAAAACATT-3'	3–21
*Msp 2*	MSP2N1 Reverse 1	5'GAGGGATGTTGCTGCTCC-3'	688–705
*Msp 2*	MSP2N2 Forward 2	5'GAGTATAAGGAGAAGTAT-3'	111–128
*Msp 2*	MSP2N2 Reverse 2	5'CTAGAACCATGCATAGTC-3'	602–620

### Gel electrophoresis

Gel electrophoresis was performed on nested PCR products to confirm the presence of PCR amplicons size of interest. Placental-cord pair, placental-infants first parasitaemia and placental-cord and infants first parasitaemia samples were run in parallel in the 2% agarose gel in 100 ml of 1 × TBE (100 mM Tris, 100 mM borate and 5 nM EDTA) solution. The gels were stained with 0.5 μg/ml ethidium bromide final concentration and the similarities and differences of the bands size was visualized by ultraviolet light. Fragment sizes were estimated using a DNA size marker (10 μl of 100 kb ladder, GeneRuler™DNA ladders). Nest two PCR products with single bands were purified by QIAquick PCR product purification kit using microcentrifuge before sequencing. For samples which presented more than one band (more than one genotype), the gel were cut along those bands that share the size, purified by QIAquick Gel extraction kit and then sequenced.

### Sequencing method

DNA sequencing was done on nest two amplicons [11]. In preparation for sequencing, excess dNTPs and unincorporated primers were eliminated from DNA by purification using QIAquick PCR purification kit (Qiagen). DNA sequencing was performed by fluorescence-based technique with an Applied Biosystems (Foster City, CA) 377 automated DNA sequencer. The purified PCR products, one prepared template were sequenced for each *msp2 *and the second template were sequenced if differences were found between the two derived sequences.

### Data analysis

Data were analysed using STARTVIEW statistical package. Descriptive analysis used to evaluate the means of the different variables. Paired t- tests were used to analyse and determine significance relationship between difference observed variables. Regression plots that showed the variability around means of different variables and *P *value < 0.05 in the analysis was considered to be significant. Chi-square test was used to determine the proportional of the placental parasitaemia among gravity (primigravid, secundigravid and multigravid). A DNASTAR Program was used to analyse the sequencing results and all results were compared with *msp2 *sequences in *Plasmodium *genome resources or *Plasmodium *database (PlasmoDB) by Basic Logical Alignment Search Tool (BLAST) so as to determine if each individual sequence matched with concordant *msp2 *gene of *P. falciparum *sequences previously deposited.

## Results

### Study population

By using PCR, placenta malaria was identified in 95 out of 100 microscopy-positive mothers and out of these 40 (42.1%) were primigravidae, 31 (34.7%) secundigravidae and 24 (25.2%) multigravidae. The mean weight of 95 babies born from placental malaria was 2.9kg with the range of 2–4.3 kg and 14.9% babies were below average birth weight while the mean gestation age was 36.3 weeks (range; 32–40 weeks). Seventy-six out of 95 (79.8%) infants were born before the normal gestation age. Infants born from mothers diagnosed with placental malaria during delivery, presented with the first clinical malaria at the mean age of 11 weeks (range; 8–64) weeks.

The prevalence of *P. falciparum *infection in placental, cord blood and infant's peripheral blood at first parasitaemia as diagnosed by microscopy and PCR are presented in Table 2. However, five (0.5%) of placental blood samples, which were positive by blood smear were negative by PCR regardless of repeat procedure. Twenty-three placental and five infant's peripheral blood sample had multiple infections (more than one strain of *msp2 *genes).

**Table 2:** Prevalence of *P. falciparum *infection in the placental, cord and infant's first parasitaemia as diagnosed by microscopy and PCR

Sample	Microscopy	PCR
Placental sample	9.8 % (100/1,022)	95 % (95/100)
Cord sample	0.4 % (4/1,022)	61% (61/100)
Infants	19.1 % (18/100)	19.1 % (18/100)

### Sequencing

Fourteen pairs of placental and cord blood samples that shared the same band size following PCR were sequenced and analysed using DNASTAR program. Sequenced samples were paired based on similarities using Megalign program. Eight (60%) out of 14 pairs were genetically related while six pairs (40%) were genetically unrelated by the difference of few nucleotides estimated at 10%. In addition, seven pairs of infant's samples at first parasitaemia and corresponding placental samples shared same band size by PCR and were sequenced. Upon megalignment, one pair (14.3%) was genetically related by 100% while six pairs (85.7%) were genetically unrelated.

Comparisons of *P. falciparum *genotypes between and within pairs were also made. The comparison was between PCR results by looking at the bands which shared the same sizes, and sequencing results through alignment of the sequences in the phylogenetic tree. Some of the *msp2 *genes observed to be sharing the band size by PCR, had sequences not closely aligned in the tree. Nevertheless, pairs that were genetically related or closely genetically related by PCR were closely arranged in the tree leaves. Analysis from *Plasmodium *database (PlasmoDB) by Basic Logical Alignment Search Tool (BLAST) indicated that all samples originated from the *msp2 *gene of *P. falciparum*.

### Placental malaria outcome

**Figure 1:**
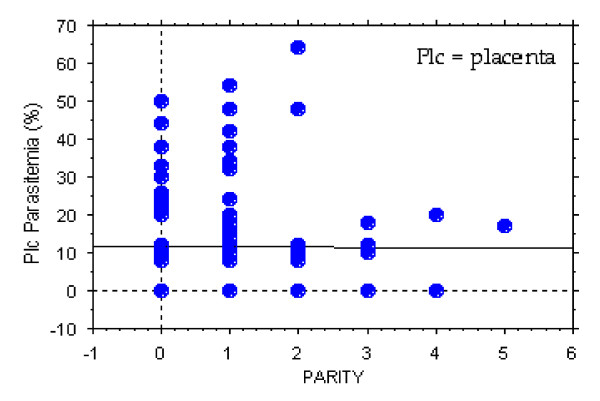
The prevalence of placental malaria which lead to symptomatic first parasitaemia malaria significantly decreased as the gravidity increased.

**Figure 2:**
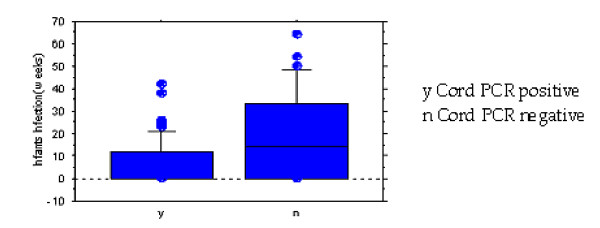
Infants presenting first parasitaemia in relation to cord malaria as diagnosed by PCR.

The prevalence of placental malaria which lead to symptomatic first parasitaemia significantly decreased as the gravidity increased (Regression coefficients, *t *= 4.8, *P *< 0.001) (Figure 1). Infants born from primigravidae were significantly more likely to be infected with *P. falciparum *(regression coefficients, *t *= 5.5, *P *< 0.001) as compared to infants born from multigravidae. It was observed that 8 out of 18 (44.4%) infants that presented with clinical malaria within three months after birth were from multigravidae mothers. Infants diagnosed with cord malaria by PCR were observed to be more susceptible to *P. falciparum *infection in their early life as compared to the infants that were cord negative, with the relative risk (RR) of 2.6 (Figure 2).

## Discussion

Malaria during pregnancy may results in foetal exposure when malaria parasites are transmitted across the placenta. In this study, prevalence of cord malaria as diagnosed by PCR was 6%. This result differs with findings by Kassberger [12], who reported 17 of the 37 (46%) of cord blood samples to be positive by PCR, while Motoi [13] diagnosed cord malaria in one baby (0.33%) by PCR, but not detected by blood smear. Prevalence differences between these studies could be due to confounding factors like geographical location. Studies in Africa suggest that 7–10% of newborn may have malaria in their cord blood though may not show evidence of active infection. High number of PCR positivity in the cord blood samples can be explained by the high sensitivity of the PCR technique [14,15], compared to microscopy. In additional, malaria parasites macromolecules and not live parasites cross the placenta and may be detected by PCR [12].

Surprisingly in this study, five of placental samples which were positive by microscopy were negative by PCR. Possible explanations include low concentrations of DNA in the placental samples, presence of point mutations at the positions where the *msp2 *specific primers anneal and the presence of haemoglobin in the DNA extracts [12].

The *msp 2 *gene can share PCR band sizes but is not an indication that the two parasites are necessarily genetically related. The sequencing results indicated that the diversity occurred due to the existence of clonal diversity in the study population. The differences between *msp2 *of *P. falciparum *parasites in the population of the analysed samples (observed between and within placental, cords blood and infants first parasitaemia) may be explained by the structural diversity in the *msp2 *gene, which is due to the central domain containing repeats that vary in number, length and sequence as previously proposed [16-19].

More than one *P. falciparum *parasite strain (multiple infections) may sequester in the placenta while only one strain crosses the placental and can be detected in the cord blood [12,20,21]. This may partly be explain the observation of placental and cord blood sample pairs that were genetically unrelated upon sequencing. The described method of cord blood sample collection was designed to minimize possible cord blood contamination with placental blood and its unlikely that cord blood contamination had significantly contributed to the observation which indicates that at least one strain from the presented complex infection in the placenta crossed to the cord. The stage of pregnancy at which parasites cross the placenta is unknown. Therefore, the majority of pairs (placental and infants), which were generically unrelated, may be partly or wholly attributed to *P. falciparum *population from the placenta to the cord, which causes clinical malaria in infants.

The findings from this study confirm that the parasite were congenitally acquired from the placenta suggesting that although neonates are thought to be immune to malaria, a proportional of them do get the disease as a result of transplacental transmission. Some explanation to the absence of parasites in the new-born includes the failure of parasites to grow in cord blood and fast elimination of parasites from the foetal circulation [22,23].

Findings from this study and others [24] suggest that placental malaria especially for primigravid may increase malaria risk in offspring. Overall, offspring of placental malaria positive mothers and infants diagnosed with cord malaria experienced their first parasitaemia at a significantly younger age. The risk of early first parasitaemia is highest among offspring of multigravid. This concurs with the findings from a previous study by Mutabingwa [24]. The reason may be that among primigravidae, the pronounced inflammatory response to placental malaria could confer protection by reducing parasitaemia [25]. In Malawi, high placental parasite densities were associated with increased risk of cord blood parasitaemia [4], supporting the conjuncture that congenital malaria could account for the relationship between placental malaria and susceptibility of infants [26]. In this study, more primigravidae were infected with malaria parasites than multigravidae, indicating the former to be at a higher risk than the later group. These observations are consistent with the findings of previous studies in malaria- endemic regions where among several factors gravidity independently influenced the occurrence of placental malaria [27,28]. It has been suggested that multigravid mothers develop malaria antibodies, which block adhesion of parasites to chondroitin sulphate A receptors in the placenta in subsequent pregnancies [29].

Placental malaria has been observed to have a negative outcome to the newborn. We observed similar negative outcome in this study where the mean gestation age were below the normal range (37 – 40) weeks in most infants born from mothers with placental malaria [30]. These findings suggest that women who had pre-term delivery could have been due to parasitization of placenta. The observation is consistent with the findings of previous studies [28,31]. However, some studies have reported conflicting results [28]. Low birth weight was associated with parasitization of the placenta. A similar association was observed elsewhere in malaria endemic sub-Saharan Africa [1,32]. The mean birth weight and low birth weight in this study were higher than those reported previously by other investigators [27,28,33]. Differences in these reports could be due to several factors, such as intensity of infection, proportion of primigravidae and or severity of infection, maternal nutrition are also important causes of low birth weight [34].

## Conclusion

In conclusion, transplacental transmission of *P. falciparum *that may lead to congenital malaria does occur as previously reported. The occurrence has important consequences for foetal and newborn development. Direct infection to the foetus could contribute to prematurity, low birth weight or increase likelihood of early and subsequent infant infections. However, the genetic unrelatedness of *P. falciparum*, which was detected in the placenta and infants below three months of age, signifies a rarity of congenital malaria and could be associated with the occurrence of maternal malaria. Further investigation of these relationships with assessment of other potential confounders, as well as their impact on infant's mortality and morbidity need to be done. Malaria prevention measures in pregnant women such as insecticide-treated nets and intermittent presumptive treatment should be a priority for malaria control programmes to prevent low birth weight, anaemia, intrauterine growth retardation, premature delivery and neonatal malaria.

## Authors' contributions

GM involved in developing the research proposal, conducting the genotyping study, analysing data, alignment of the sequenced results and writing the initial and the final manuscript. SK participated in designing of the study and analysis of the research results. LB assisted in writing the initial and final manuscript. All authors have seen and approved the final manuscript and are in agreement with my being the corresponding author.
